# Expression of genes related to lipid transport in meat-type ducks divergent for low or high residual feed intake

**DOI:** 10.5713/ajas.19.0284

**Published:** 2019-08-26

**Authors:** Sihua Jin, Yuan Xu, He Zang, Lei Yang, Zhiqiang Lin, Yongsheng Li, Zhaoyu Geng

**Affiliations:** 1 College of Animal Science and Technology, Anhui Agricultural University, Hefei 230036, China; 2 Anhui Provincial Key Laboratory of Local Animal Genetic Resources Conservation and Bio-breeding, Hefei 230036, China; 3 Huangshan Qiangying Duck Breeding Co. Ltd., Huangshan 245461, China

**Keywords:** Residual Feed Intake, Lipid Transport, Albumin (*ALB*), Fatty Acid Hydroxylase Domain Containing 2 (*FAXDC2*), Association, Meat-type Ducks

## Abstract

**Objective:**

This study examined the effects of divergence in residual feed intake (RFI) on expression profiles of key genes related to lipid transport in the liver and duodenal epithelium and their associations with feed efficiency traits in meat-type ducks.

**Methods:**

A total of 1,000 male ducks with similar body weight (1,042.1±87.2 g) were used in this study, and their individual RFI was calculated from 21 to 42 d of age. Finally, the 10 highest RFI (HRFI) and 10 lowest RFI (LRFI) ducks were chosen for examining the expression of key genes related to lipid transport in the liver and duodenal epithelium using quantitative polymerase chain reaction.

**Results:**

In the liver, expression levels of albumin (*ALB*), *CD36* molecule (*CD36*), fatty acid hydroxylase domain containing 2 (*FAXDC2*), and choline kinase alpha (*CHKA*) were significantly higher in LRFI ducks than in HRFI ducks (p<0.01); negative correlations (p<0.05) between expression levels of *ALB*, *CD36*, *FAXDC2*, and *CHKA* and RFI were detected in the liver. Additionally, *ALB* expression was strongly positively correlated (p<0.05) with *CD36*, *FAXDC2*, *CHKA*, and apolipoprotein H (*APOH*) expression in the liver. In duodenal epithelium, we found that mRNA levels of *ALB*, *CD36*, *FAXDC2*, and *APOH* were significantly higher in LRFI ducks than in HRFI ducks (p<0.01); RFI was strongly negatively correlated (p<0.05) with *ALB*, *FAXDC2*, and *APOH* expression, while *ALB* expression was strongly positively correlated with *APOH* expression (p<0.01) in duodenal epithelium. Furthermore, expression levels of both *ALB* and *FAXDC2* genes were significantly associated with feed conversion ratio and RFI in both liver and duodenal epithelium (p<0.05).

**Conclusion:**

Our findings therefore suggest that *ALB* and *FAXDC2* genes might be used as potential gene markers designed to improve feed efficiency in future meat-type duck breeding programs.

## INTRODUCTION

Feed costs account for about 70% of the total cost of the modern poultry industry. Therefore, improving feed efficiency is a crucial breeding target in poultry production. Residual feed intake (RFI) is an important index in assessing feed efficiency traits in poultry [1,2]. The RFI was first proposed by Koch [3] in 1963, and it is used to describe the difference between observed feed intake and predicted feed intake. Moreover, RFI is a suitable index for breeding animals because selection based on RFI improves animals’ feed efficiency without impairing production performance during the growing period [4]. Furthermore, studying the potential candidate genes and genetic markers of feed efficiency is conducive to reducing both feed cost and nitrogen emissions [5].

An earlier transcriptomic analysis in chickens showed that the gene expression difference between high RFI (HRFI) and low RFI (LRFI) animals can be explained by differences in cell division, growth, proliferation and apoptosis, protein synthesis, lipid metabolism, and transport of cellular molecules [6]. Particularly, components of lipid metabolism, including fatty acid profiles, lipid transport, lipid oxidation, and enzyme activities affect feed efficiency traits [7]. Lipid transport plays an especially crucial role in lipid metabolism and involves two opposite biological processes in tissues: loading, involving energy utilization and storage, and unloading, involving endogenous synthesis and lipid release [8]. A previous study using RNA-Seq showed that broiler chickens with low feed efficiency increased expression of genes related to lipid synthesis and reduced expression of genes related to lipid transport (i.e. triglyceride and cholesterol) in the abdominal fat tissue using RNA-Seq [9].

The liver and duodenum both play vital roles in the regu lation of lipid absorption, metabolism, and appetite regulation [10,11]. The liver is a key organ in lipid processing, packaging, and redistribution [12]. Interestingly, *de novo* biosynthesis of fatty acids mainly occurs in the liver (more than 70%) rather than in the adipose tissue in birds, unlike in mammals. In addition, the liver secretes bile that is transferred to the duodenum, where it emulsifies fat and promotes fat decomposition and absorption [13]. Moreover, the duodenum is the segment of the gastrointestinal tract and plays a key role in lipid digestion and transport [14]. The duodenum accepts pancreatic juice secreted by the pancreas and bile inflow from the gallbladder for lipid digestion when partially digested food enters the stomach [15]. The duodenum also plays an important role in regulating appetite through gut nutrient chemosensors besides helping in digestion [16]. Furthermore, a global view of gene expression in divergent RFI chickens showed that genes involved in the metabolism and transport of lipid, cholesterol, and bile were upregulated in the duodenum of LRFI chickens [6]. Hence, it is reasonable to propose that the expression of genes related to lipid transport in the liver and duodenum might influence the regulation of feed efficiency traits.

Until now, there are fewer studies on the associations of the expression of key genes related to lipid transport with feed efficiency traits in meat-type ducks. Therefore, this study was conducted to investigate the expression profiles of key genes related to lipid transport, including angiotensin I converting enzyme (*ACE*), albumin (*ALB*), CD36 molecule (*CD36*), fatty acid hydroxylase domain containing 2 (*FAXDC2*), choline kinase alpha (*CHKA*), and apolipoprotein H (*APOH*) in the liver and duodenal epithelium of meat-type ducks divergent for RFI, which aimed to evaluate the relationship of these genes expression with feed efficiency traits in meat-type ducks. Our study is expected to provide more information for breeders and farmers to allow the selection of high feed efficiency ducks in future meat-type duck breeding programs.

## MATERIALS AND METHODS

### Ethics statement

All animal experiments were carried out in accordance with guidelines of the Ministry of Science and Technology (revised in 2004, Beijing, China). The care and use of all ducks in the current experiment was authorized and endorsed by the Animal Care and Use Committee of Anhui Agricultural University (approval number: SYXK 2016-007), and all efforts were made to minimize suffering of the ducks used.

### Experimental design and animal husbandry

All meat-type ducks used in this study were provided and raised by Huangshan Qiangying Duck Breeding Co., Ltd, Huangshan, China. A total of 2,200 male ducks were randomly selected from a random mating population, which were from genetically unrelated sources and selected as important sources for future meat-type duck breeding programs based on appearance, feed efficiency, and slaughter traits in each generation. The experimental ducks were of the sixth generation and were pedigreed by mating 200 male with 1,000 female ducks in two hatches. All ducks were sexed, wing-banded, and weighed on the hatching day and raised on the floor for the first two weeks. At 21 d of age, a total of 1,000 ducks with similar body weight (1,042.1±87.2 g) were transferred to individual cages with the size of 55 cm×50 cm ×40 cm. All experimental ducks were reared in the same house with the same lighting schedule and management procedure according to the company’s management guidelines. Feed intake of each duck was recorded in the individual cages, and the same basal diet (Table 1) and water were offered *ad libitum* during the entire period. All ducks were exposed to continuous illumination (24 L:0 D) for the first three days after hatching, and then lighting regime of 20 L:4 D was used during the experimental period. At 42 d of age, all ducks were fasted for 12 h and then their body weight was measured and recorded. The average daily feed intake (ADFI), metabolic body weight (MBW^0.75^), and average daily weight gain (ADG) during the experimental period of individual birds were determined, and the individual RFI and feed conversion ratio (FCR) were calculated. Regression analysis was performed for each bird and a mixed model was used to estimate duck RFI values [1] using SAS version 9.4 (SAS Institute Inc., Cary, NC, USA) according to the following equation:

$$\text{RFI}=\text{ADFI}-(b_{0}+b_{1}\times {\text{MBW}}^{0.75}+b_{2}\times \text{ADG})$$

where *b_0_* is the intercept and *b_1_* and *b_2_* are of partial regression coefficients of MBW^0.75^ and ADG on ADFI, respectively.

### Sample selection and tissue harvest

After eliminating outlier data, the RFI value for each duck was calculated. Moreover, we selected the highest (n = 30, inefficient) and lowest (n = 30, efficient) RFI ranking ducks to optimize the samples since the mean RFI rank was subjected to outlier or extreme values. At 42 d of age, the 10 HRFI and 10 LRFI ducks were finally selected to represent two divergent RFI groups. After exsanguination, liver samples were immediately collected from similar liver sites of each bird, and then the whole gastrointestinal tract was taken out after dissecting the abdominal cavity. Duodenal tissue (5 cm long) was harvested approximately 5 cm distal to the abomasal-duodenal junction and then washed with sterile phosphate buffered saline (PBS). Epithelial tissue samples of the duodenum were then scraped from the underlying connective and muscular tissues using the back of a surgical knife, after which the tissue was washed with PBS. All tissue samples were transferred immediately to RNALater (ThermoFisher Scientific, Santa Clara, CA, USA) and subsequently stored at −80°C until RNA extraction.

### Total RNA extraction and quantitative polymerase chain reaction

Total RNA was extracted from the liver (30.0 mg) and duodenal epithelium (40.0 mg) using Bizol RNA kit (Biomiga, San Diego, CA, USA) according to the manufacturer’s instructions. The quantity of RNA was determined by measuring the absorbance at 260, 280, and 230 nm using a NanoDrop 2000 spectrophotometer (ThermoFisher Scientific, USA), followed by assessment of integrity through agarose gel electrophoresis. Only RNA 260/280 absorbance ratios between 1.8 and 2.1 were considered pure and were used for further analysis.

Total RNA (1.0 μg) were reverse transcribed into cDNA using PrimerScript RT reagent kit (TaKaRa, Otsu Shiga, Japan) according to the manufacturer’s instructions. The mRNA gene sequences related to lipid transport were obtained from the GenBank database (http://www.ncbi.nlm.nih.gov), and primer pairs of target genes were designed by Primer Premier 5.0 software (Premier Biosoft International, Palo Alto, CA, USA). Primer sequences are shown in Table 2. All primers were synthesized by the Beijing Tsingke Biological Technology Co. Ltd., Beijing, China.

The quantitative polymerase chain reaction (qPCR) reac tion volume was 20.0 μL, which included 1.0 μL gene-specific primer, 8.0 μL ddH_2_O, 10.0 μL 2×SYBR Green Master Mix (Applied Biosystems, Foster City, CA, USA) and 1.0 μL cDNA (obtained from the liver or duodenal epithelium). The thermal cycling program constituted the following steps: 95°C for 5 min, 40 cycles of 95°C for 15 s and 60°C for 1 min, 95°C for 15 s, 60°C for 1 min, 95°C for 15 s, and 60°C for 15 s. All qPCR reactions were conducted in triplicate and a no-template control was on each plate of an ABI 7500 PCR apparatus (ThermoFisher Scientific, USA), and then gel electrophoresis was performed after PCR amplification to ensure primer specificity and a single PCR product. Finally, the average cycle threshold (Ct) values after calibrating to *β-actin* were used for calculating the relative expression of each target gene using the 2^−ΔΔCt^ method [17].

### Statistical analysis

The average cycle threshold (Ct) values were calculated using the SAS version 9.4 (SAS Institute Inc., USA). The expression level of each target gene was generated with reference to *β-actin* housekeeping genes. All data were checked for quantile-quantile plots, normality, and homogeneity of variance by generating histograms and through formal statistical tests as part of the UNIVARIATE procedure of SAS 9.4. Measurements followed a completely randomized design, and data were analyzed using the general linear model procedure of SAS 9.4. The difference of gene expression and phenotypic data between the two RFI groups were analyzed using Student’s *t*-test of SAS 9.4. The correlation between the expression of individual genes, and between gene expression and feed efficiency traits were calculated by estimating Pearson’s product-moment correlation using PROC CORR of SAS 9.4. All data shown in tables are expressed as mean±standard deviation (SD). Differences were considered statistically significant at p<0.05.

## RESULTS

### Comparison of feed efficiency traits in ducks with different residual feed intake

The details of phenotypic traits have been displayed in Table 3. Briefly, the two experimental groups were significantly different in terms of FCR, RFI, and ADFI (p<0.05); the HRFI group consumed 9.46% more feed than did the LRFI group. There was no significant difference in terms of ADG and MBW^0.75^ between the HRFI and LRFI ducks (p>0.05).

### Expression of genes related to lipid transport in the liver and duodenal epithelium

**Figure 1:**
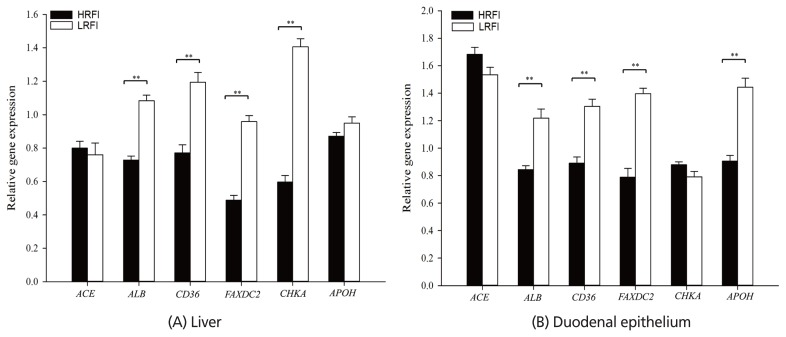
Differential expression of genes related to lipid transport in liver (A) and duodenal epithelium (B) of meat-type ducks divergent for residual feed intake (RFI). * p<0.05, ** p<0.01.

The relative expression of key genes related to lipid transport in the liver and duodenal epithelium have been displayed in Figure 1. In the liver, the expression levels of *ALB* and *CD36* were significantly higher in the LRFI ducks than in the HRFI ducks (p<0.01), and the expression levels of *FAXDC2* and *CHKA* were approximately twofold higher in the LRFI ducks than in the HRFI ducks (p<0.01). There was no significant difference in the expression levels of *ACE* and *APOH* between the HRFI and LRFI ducks.

In the duodenal epithelium of the LRFI ducks, the mRNA levels of *ALB*, *CD36*, *FAXDC2*, and *APOH* were significantly upregulated when compared with the HRFI ducks (p<0.01), and there was no significant difference in *ACE* and *CHKA* mRNA levels between the HRFI and LRFI ducks.

### Correlation of feed efficiency traits with genes related to lipid transport

The correlation coefficients for feed efficiency traits and expression levels of genes related to lipid transport in the liver and duodenal epithelium are displayed in Tables 4, 5, respectively. In the liver, there was a strong negative correlation (ranging from r = −0.75 to −0.97; p<0.05) between the expression of *ALB*, *CD36*, *FAXDC2*, *CHKA*, and FCR. Furthermore, RFI was strongly and negatively correlated (ranging from r = −0.86 to −0.94, p<0.01) with *FAXDC2* and *CHKA*, while it showed moderate negative correlation with *ALB* (r = −0.73; p<0.05) and *CD36* (r = −0.63; p<0.05). Moreover, ADFI was strongly and negatively correlated with *CHKA* (r = −0.80; p<0.05). ADG was strongly and positively correlated with *ALB* (r = 0.87; p<0.01). MBW^0.75^ was strongly and negatively correlated with *CHKA* (r = −0.80; p<0.05).

In the duodenal epithelium, FCR was strongly and negatively correlated (ranging from r = −0.78 to −0.87; p<0.05) with *ALB*, *FAXDC2*, and *APOH*, while RFI was strongly and negatively correlated (ranging from r = −0.74 to −0.84, p<0.05) with *ALB*, *FAXDC2*, and *APOH*. In addition, ADFI was strongly and negatively correlated with *FAXDC2* (r = −0.76; p<0.05).

### Correlation between the expressions of genes related to lipid transport

Correlation coefficients between the expression of genes related to lipid transport in the liver and duodenal epithelium have been reported in Tables 6, 7, respectively. In the liver, the *ALB* expression was strongly and positively correlated (ranging from r = 0.63 to 0.93, p<0.05) with *CD36*, *FAXDC2*, *CHKA*, and *APOH* expression. Therein, *CHKA* expression was strongly and positively correlated (ranging from r = 0.83 to 0.96, p<0.05) with *CD36* and *FAXDC2* expression. Furthermore, *CD36* expression was strongly and positively correlated with *FAXDC2* expression (r = 0.86; p<0.05).

In the duodenal epithelium, *APOH* expression was strongly and positively correlated with *ALB* expression (r = 0.73; p< 0.01), and moderately and positively correlated with *FAXDC2* expression (r = 0.58; p<0.05). In addition, *CHKA* expression was moderately and positively correlated with *ALB* expression (r = 0.50; p<0.05).

## DISCUSSION

In recent years, RFI has become an important parameter for assessing feed efficiency in meat-type ducks [2]. In the present study, the HRFI ducks consumed more feed than did the LRFI ducks, while there was no significant difference between them in terms of ADG. This result was consistent with the findings of a previous study on chickens [18], and it may reflect that LRFI (efficient) animals consume less feed without experiencing significant changes in growth performance when compared with HRFI (inefficient) animals.

It is well documented that components of lipid metabo lism, such as triacylglycerol transport and lipid accumulation in the liver and duodenum, may be involved in the regulation of RFI in cattle [19]. Recently, a global transcriptome analysis in the duodenum of laying ducks showed that genes involved in lipid metabolism play an important role in regulating feed efficiency [20]. Moreover, a previous study suggested that lipid metabolism in the liver and lipid absorption in the intestine were two important factors affecting feed efficiency traits [10]. Lipids are a vital nutrient widely utilized in almost all tissues of the body, and they play a key role in animal growth and development [21]. However, lipid transport requires special protein transporters in circulation (blood) because lipids are water-insoluble and amphiphilic [7,22]. Hence, in the present study, we first focused on genes encoding lipid transporters, including *ALB*, *CHKA*, and *APOH*. Of these, *ALB* encodes serum albumin, which contributes to blood plasma colloid osmotic pressure and plays a key role in transporting metabolites such as fatty acids and hormones in blood vessels [23]. In this study, *ALB* was significantly upregulated in the liver and duodenal epithelium of LRFI ducks, and it was strongly and negatively correlated with FCR and RFI. This result might be ascribed to the upregulation of *ALB*, thereby increasing the level of albumin in the blood, which may further enhance the transport of fatty acid and other lipid molecules.

In addition, *CHKA* and *APOH* are genes encoding two other special protein transporters of lipid [6]. *CHKA* plays an important role in phospholipid biosynthesis via the CDP-choline pathway [24]. *APOH*, also called β2 glycoprotein I, may be involved in triglyceride-rich lipoprotein clearance by activation of lipoprotein lipase [25]. In this study, *CHKA* was significantly upregulated in the liver of LRFI ducks, and *APOH* was significantly upregulated in the duodenal epithelium of LRFI ducks. Additionally, *CHKA* and *APOH* were also strongly and positively correlated with *ALB*. Taken together, these data suggest that LRFI ducks have upregulated expression of genes encoding lipid transporters, such as *ALB*, *CHKA*, and *APOH*, in the duodenal epithelium, when compared with the HRFI ducks. This was consistent with the results of a previous transcriptomic analysis of the duodenum of meat-type chickens with divergent RFI [6]. In agreement with the findings of our current study, it was demonstrated that the LRFI cattle had upregulation of genes involved in molecular transport and reduced hepatic lipid accumulation relative to the HRFI cattle [26]. On the contrary, this result may reflect that LRFI ducks tend to scavenge available fatty acids from the liver and intestines.

*FAXDC2* belongs to the fatty acid hydroxylase superfamily that includes fatty acid and carotene hydroxylases and sterol desaturases, which are involved in cholesterol biosynthesis [27]. Specifically, fatty acid hydroxylase can alter the packing structures of membrane microdomains, and thus, regulates cellular function of membrane-associated proteins [28]. It is also involved in the synthesis of sphingolipids in plasma membrane rafts, which control lipid raft mobility [29]. In this study, *FAXDC2* was significantly upregulated in the liver and duodenal epithelium of LRFI ducks, and it was strongly and negatively correlated with FCR and RFI. Similarly, expression of *FAXDC2* mRNA in the duodenum was found to be significantly upregulated in LRFI chickens than in HRFI chickens [30].

*CD36* encodes the protein that is important in fatty acids recognition and fat perception, and this protein has multiple functions in different tissues via the binding of different ligands. In the liver, *CD36* plays an important role in both the uptake of fatty acids from exogenous sources and the release of lipids into circulation [31]. *CD36* deletion reduces the secretion of very low-density lipoproteins, which are involved in the transport of lipids within the liver of mice [32]. In the duodenum, *CD36* can act in gut fat absorption [33] and plays a crucial role in the absorption of long-chain fatty acids (LFA), and the LFA can bind with albumin in circulation [34]. Intestinal *CD36* is involved in postprandial lipid metabolism and promotes rapid clearance of triglyceride-rich lipoprotein in the blood [35]. Moreover, lipid binding to intestinal *CD36* could produce a satiety effect and modulate feed intake in mice [36]. Taken together, upregulation of *CD36* in the liver can promote lipid transport, upregulation of *CD36* in the duodenum, improvement of postprandial lipid transport, and enhancement of satiety, thereby decreasing feed intake. In the present study, *CD36* was significantly upregulated in the liver and duodenal epithelium of LRFI ducks; and was strongly and negatively correlated with FCR and RFI in liver. Hence, this data might indicate that LRFI ducks are more easily satiated and have enhanced postprandial lipid metabolism relative to HRFI ducks.

In summary, genes related to lipid transport were upregu lated in the liver and duodenal epithelium of LRFI ducks than in HRFI ducks, indicating that genes related to lipid transport might participate in the genetic regulatory network of feed efficiency traits in meat-type ducks. Moreover, the expression of *ALB* and *FAXDC2* were significantly correlated with FCR and RFI in the liver and duodenal epithelium, suggesting functional roles for these genes in regulating feed efficiency, which might be used as potential gene markers to improve feed efficiency in future meat-type duck breeding programs. Further studies are necessary to examine the molecular mechanisms of these differentially expressed genes on feed efficiency traits as well as possible alternations during their translation to proteins in poultry.

## Figures and Tables

**Table 1:** Compositions and calculated nutrient values of the basal diet for experimental ducks

Compositions (%)	Content
Corn	60.00
Wheat flour	8.00
Corn gluten meal	26.00
Soybean oil	2.20
Limestone	1.50
Calcium hydrogen phosphate	1.02
Sodium chloride	0.35
Lysine	0.40
Methionine	0.13
Threonine	0.10
Premix1)	0.30
Calculated nutrient values	
Metabolizable energy (kcal/kg)	3,000.00
Crude protein (%)	17.50
Nonphytate phosphorus (%)	0.36
Total phosphorus (%)	0.50
Calcium (%)	0.80
Methionine (%)	0.40
Lysine (%)	1.10
Cysteine (%)	0.29

1) Composition supplied per kilogram of diet: vitamin A, 8,000 IU; vitamin D_3_, 3,000 IU; vitamin E, 20 IU; vitamin B_1_, 1.6 mg; vitamin B_2_, 10.0 mg; vitamin B_12_, 0.02 mg; vitamin K_3_, 3.0 mg; calcium-D-pantothenate, 11.0 mg; nicotinic acid, 40.0 mg; folic acid, 0.75 mg; biotin, 0.20 mg; vitamin B_6_, 2.00 mg; choline chloride, 1,000 mg; Fe, 80 mg; Mn, 60 mg; I, 0.20 mg; Se, 0.20 mg; Zn, 60 mg; Cu, 8 mg; Cr 0.20 mg.

**Table 2:** Primers used for quantitative real-time polymerase chain reaction

Gene	Accession number	Primers	Sequences (5′-3′)	Product size (bp)
*ACE*	XM_005029298.3	Forward	CGCATCAAGGAGGACGAGTACAAC	178
		Reverse	GAACTGGAACTGGATCACGAAGC	
*ALB*	NM_001310394.1	Forward	TAAACGCAAGCCCCAGATGA	111
		Reverse	TCGCCAAAGCATGTCTCGAT	
*CD36*	XM_005016712.4	Forward	CTCGCATTCCTGGGTTCTTA	108
		Reverse	ATGCTGCTTGGCTGAAACTT	
*FAXDC2*	XM_005026187.3	Forward	CGACTCCTTTGCCTAAACCCT	129
		Reverse	GCTCTTTGCTTACCTCCTGC	
*CHKA*	XM_013094843.2	Forward	AATGACTTGCAAGGGGCAGA	110
		Reverse	AGTCGCCCTTGTGGAAAGAT	
*APOH*	XM_005016097.3	Forward	TCGTGTGAACCAGGCTACAA	104
		Reverse	GGACACGTCACAGGTTGACA	
*β-actin*	NM_001310421.1	Forward	GCAAGTACTCTGTCTGGATTGGAG	116
		Reverse	TTTGCGGTGGACAATGGA	

*ACE*, angiotensin I converting enzyme; *ALB*, albumin; *CD36*, CD36 molecule; *FAXDC2*, fatty acid hydroxylase domain containing 2; *CHKA*, choline kinase alpha; *APOH*, apolipoprotein H; *β-actin*, beta-actin.

**Table 3:** Basic statistics of feed efficiency traits between low and high RFI ducks

Traits	HRFI	LRFI
ADFI (g/d)	287.69±21.27^a^	262.83±20.25^b^
ADG (g/d)	129.53±8.99	139.5±10.36
MBW^0.75^ (g/d)	262.53±10.96	351.94±10.96
FCR (g/g)	2.22±0.02^a^	1.88±0.01^b^
RFI (g/d)	12.31±5.82^a^	−14.78±6.34^b^

RFI, residual feed intake; ADFI, average daily feed intake; ADG, average weight gain; MBW^0.75^, metabolic body weight; FCR, feed conversion ratio; HRFI, highest RFI; LRFI, lowest RFI.

The number of samples of the HRFI and LRFI groups is 10, respectively.

a,b Means within a row without a common superscript differ significantly (p<0.05).

**Table 4:** Associations of expressions of genes related to lipid transport with feed efficiency traits in liver

Gene	ADFI	ADG	MBW^0.75^	FCR	RFI
*ACE*	0.27	0.08	0.23	0.21	0.26
*ALB*	−0.17	0.87**	−0.11	−0.89**	−0.73*
*CD36*	−0.48	0.36	−0.56	−0.75**	−0.63*
*FAXDC2*	−0.63	0.27	−0.61	−0.92**	−0.86**
*CHKA*	−0.80*	0.24	−0.80*	−0.97**	−0.94**
*APOH*	−0.13	0.44	−0.18	−0.51	−0.38

ADFI, average daily feed intake; ADG, average weight gain; MBW0.75, metabolic body weight; FCR, feed conversion ratio; RFI, residual feed intake; *ACE*, angiotensin I converting enzyme; *ALB*, albumin; *CD36*, CD36 molecule; *FAXDC2*, fatty acid hydroxylase domain containing 2; *CHKA*, choline kinase alpha; *APOH*, apolipoprotein H; LRFI, lowest RFI; HRFI, highest RFI.

The number of samples of LRFI and HRFI groups is 10, respectively.

* p<0.05,

** p<0.01.

**Table 5:** Associations of expressions of genes related to lipid transport with feed efficiency traits in duodenal epithelium

Gene	ADFI	ADG	MBW^0.75^	FCR	RFI
*ACE*	0.37	−0.11	0.25	0.50	0.52
*ALB*	−0.03	0.69	0.19	−0.82*	−0.74*
*CD36*	−0.13	0.36	−0.35	−0.47	−0.23
*FAXDC2*	−0.76*	0.01	−0.53	−0.78**	−0.84**
*CHKA*	0.60	0.30	0.50	0.34	0.48
*APOH*	−0.34	0.51	−0.11	−0.87**	−0.80**

ADFI, average daily feed intake; ADG, average daily weight gain; MBW^0.75^, metabolic body weight; FCR, feed conversion ratio; RFI, residual feed intake; *ACE*, angiotensin I converting enzyme; *ALB*, albumin; *CD36*, CD36 molecule; *FAXDC2*, fatty acid hydroxylase domain containing 2; *CHKA*, choline kinase alpha; *APOH*, apolipoprotein H; LRFI, lowest RFI; HRFI, highest RFI.

The number of samples of LRFI and HRFI groups is 10, respectively.

* p<0.05,

** p<0.01.

**Table 6:** Associations between expressions of genes related to lipid transport in liver of meat-type ducks

Gene	*ACE*	*ALB*	*CD36*	*FAXDC2*	*CHKA*	*APOH*
*ACE*	1	−0.18	−0.13	0.13	−0.21	−0.32
*ALB*	-	1	0.73*	0.93**	0.88*	0.63**
*CD36*	-	-	1	0.86*	0.83*	0.45*
*FAXDC2*	-	-	-	1	0.96*	0.29
*CHKA*	-	-	-	-	1	0.84**
*APOH*	-	-	-	-	-	1

*ACE*, angiotensin I converting enzyme; *ALB*, albumin; *CD36*, CD36 molecule; *FAXDC2*, fatty acid hydroxylase domain containing 2; *CHKA*, choline kinase alpha; *APOH*, apolipoprotein H.

The sample of number of highest residual feed intake and lowest residual feed intake groups is 10, respectively.

* p<0.05,

** p<0.01.

**Table 7:** Associations between expressions of genes related to lipid transport in duodenal epithelium of meat-type ducks

Gene	*ACE*	*ALB*	*CD36*	*FAXDC2*	*CHKA*	*APOH*
*ACE*	1	−0.03	−0.16	−0.30	0.13	0.01
*ALB*	-	1	0.33	0.35	0.50*	0.73**
*CD36*	-	-	1	−0.16	0.05	0.36
*FAXDC2*	-	-	-	1	−0.33	0.58*
*CHKA*	-	-	-	-	1	−0.39
*APOH*	-	-	-	-	-	1

*ACE*, angiotensin I converting enzyme; *ALB*, albumin; *CD36*, CD36 molecule; *FAXDC2*, fatty acid hydroxylase domain containing 2; *CHKA*, choline kinase alpha; *APOH*, apolipoprotein H.

The sample of number of highest residual feed intake and lowest residual feed intake groups is 10, respectively.

* p<0.05,

** p<0.01.
